# Changes in serum DAMPs and cytokines/chemokines during near‐infrared photoimmunotherapy for patients with head and neck cancer

**DOI:** 10.1002/cam4.6863

**Published:** 2023-12-22

**Authors:** Hiromasa Ishihara, Daisuke Nishikawa, Daisuke Muraoka, Katsuhiro Masago, Shintaro Beppu, Hoshino Terada, Hirokazu Matsushita, Nobuhiro Hanai

**Affiliations:** ^1^ Division of Translational Oncoimmunology Aichi Cancer Center Research Institute Nagoya Japan; ^2^ Department of Head and Neck Surgery Aichi Cancer Center Hospital Nagoya Japan; ^3^ Department of Pathology and Molecular Diagnostics Aichi Cancer Center Hospital Nagoya Japan; ^4^ Division of Cancer Immunogenomics Nagoya University Graduate School of Medicine Nagoya Japan

**Keywords:** cytokine/chemokine, head and neck cancer, immunogenic cell death, near‐infrared photoimmunotherapy, neutrophil‐to‐lymphocyte ratio

## Abstract

**Background:**

Near‐infrared photoimmunotherapy (NIR‐PIT) for head and neck cancer is a recently developed therapy. However, there is limited data on patients receiving NIR‐PIT in real clinical settings.

**Methods:**

Seven NIR‐PIT sessions were administered to five patients with head and neck squamous cell carcinoma (HNSCC). Serum damage‐associated molecular patterns (DAMPs) (HMGB1 and Hsp70 levels), and cytokine and chemokine production, were compared before and after NIR‐PIT.

**Results:**

The serum concentration of HMGB1 increased after NIR‐PIT (*p* = 0.031, Wilcoxon test) in all patients except one who did not achieve a clinical response. Chemokines MIP‐1α (CCL3) and MIP‐1β (CCL4) increased significantly 1–3 days after treatment (CCL3, *p* = 0.0036; CCL4, *p* = 0.0016, Wilcoxon test). A low pre‐treatment neutrophil‐to‐lymphocyte ratio (NLR) was associated with a better response to therapy and survival.

**Conclusions:**

The release of DAMPs, and cytokine/chemokine production, were detected in the patients' peripheral blood. The baseline NLR may predict patient outcomes in response to NIR‐PIT.

## 1 INTRODUCTION

Head and neck squamous cell carcinoma (HNSCC) is the sixth most common cancer worldwide.^1^ The current standard of care for unresectable recurrent or metastatic HNSCC is chemotherapy or chemotherapy plus EGFR‐targeted therapy (cetuximab) and treatment with immune checkpoint inhibitors (ICIs).^2^, ^3^ However, even in cases of local disease, the potential for cure with these therapies is currently limited.^4^, ^5^ Therefore, there is an urgent clinical need for novel therapies for recurrent or metastatic HNSCC that can control local disease without causing severe functional disability.

NIR‐PIT is a recently developed and highly selective two‐step method of cancer treatment. First, a monoclonal antibody conjugated to the light‐absorbing dye IRDye700DX (IR700) is intravenously injected. Each antibody‐photoabsorbent conjugate (APC) binds to its cognate receptor that is overexpressed on the surface of cancer cells. Second, a laser delivers visible‐near‐infrared light to excite IR700 in the APCs, causing rapid, highly selective, and lethal damage to the cell membranes.^6^ Thus, NIR‐PIT can be applied to any cancer with overexpressed target membrane proteins for which there is a suitable monoclonal antibody.^7^

Phase I/II trials of NIR‐PIT using cetuximab‐IR700 targeting EGFR in patients with inoperable HNSCC revealed that NIR‐PIT was well‐tolerated.^8^ A domestic Phase I study in Japan also showed good tolerability.^9^ Although international phase III trials are ongoing, NIR‐PIT was conditionally approved by the Japanese Pharmaceuticals and Medical Devices Agency (PMDA) in 2020 and has been in clinical use for patients with unresectable locally advanced disease since January 2021.^10^

NIR‐PIT treatment has been shown to induce immunogenic cell death (ICD) within minutes, as targeted cancer cells rupture and leak their cytoplasmic contents.^11^ Therefore, damage‐associated molecular patterns (DAMPs), endogenous danger molecules, are released from damaged or dying cells into the extracellular environment, resulting in the activation of innate immune cells.^12^, ^13^ However, these phenomena have not been reported in patients who have received NIR‐PIT in clinical settings. In this study, we used pre‐ and post‐treatment peripheral blood samples from patients treated with NIR‐PIT to investigate changes in DAMPs, production of cytokines and chemokines, and their relationship with treatment efficacy and adverse events. In addition, we evaluated the relationship between baseline immunological status and patient outcomes.

## 2 METHODS

### 2.1 Patients and sample collection

Five patients with HNSCC underwent seven NIR‐PIT sessions at the Aichi Cancer Center Hospital between November 2021 and October 2022. Serum samples were obtained 1 day before and 1–3 days after treatment. Patient HN003 received the treatment three times with an interval of at least 4 weeks between cycles. All procedures were in accordance with the ethical standards of the institutions and with the 1964 Helsinki Declaration and its later amendments or comparable ethical standards. Written informed consent was obtained from all individual participants included in the study.

### 2.2 ELISA

According to the manufacturer's instructions, serum HMGB1 and Hsp70 concentrations were measured using ELISA kits from Shino‐Test Corp. (Sagamihara‐shi, Japan) and Proteintech (Tokyo, Japan), respectively.

### 2.3 Multiplex cytokine and chemokine assay

ProcartaPlex™ multiplex immunoassay was applied for cytokine and chemokine analysis using the Luminex® 100/200™ system (Luminex Corporation, Austin, TX, USA). Serum samples were tested with a commercially available panel: Th1/Th2 Cytokine & Chemokine 20‐Plex Panel 1 (Thermo Fisher Scientific, Waltham, MA, USA). All samples were processed according to the manufacturer's instructions.

### 2.4 Statistical analysis

All clinical data were analyzed using the R V.4.2.3 (R Core Team, Vienna, Austria). A paired Wilcoxon nonparametric test was used to compare the results. A *p* < 0.05 was considered statistically significant.

## 3 RESULTS

### 3.1 Increased levels of DAMPs in sera after NIR‐PIT

**FIGURE 1:**
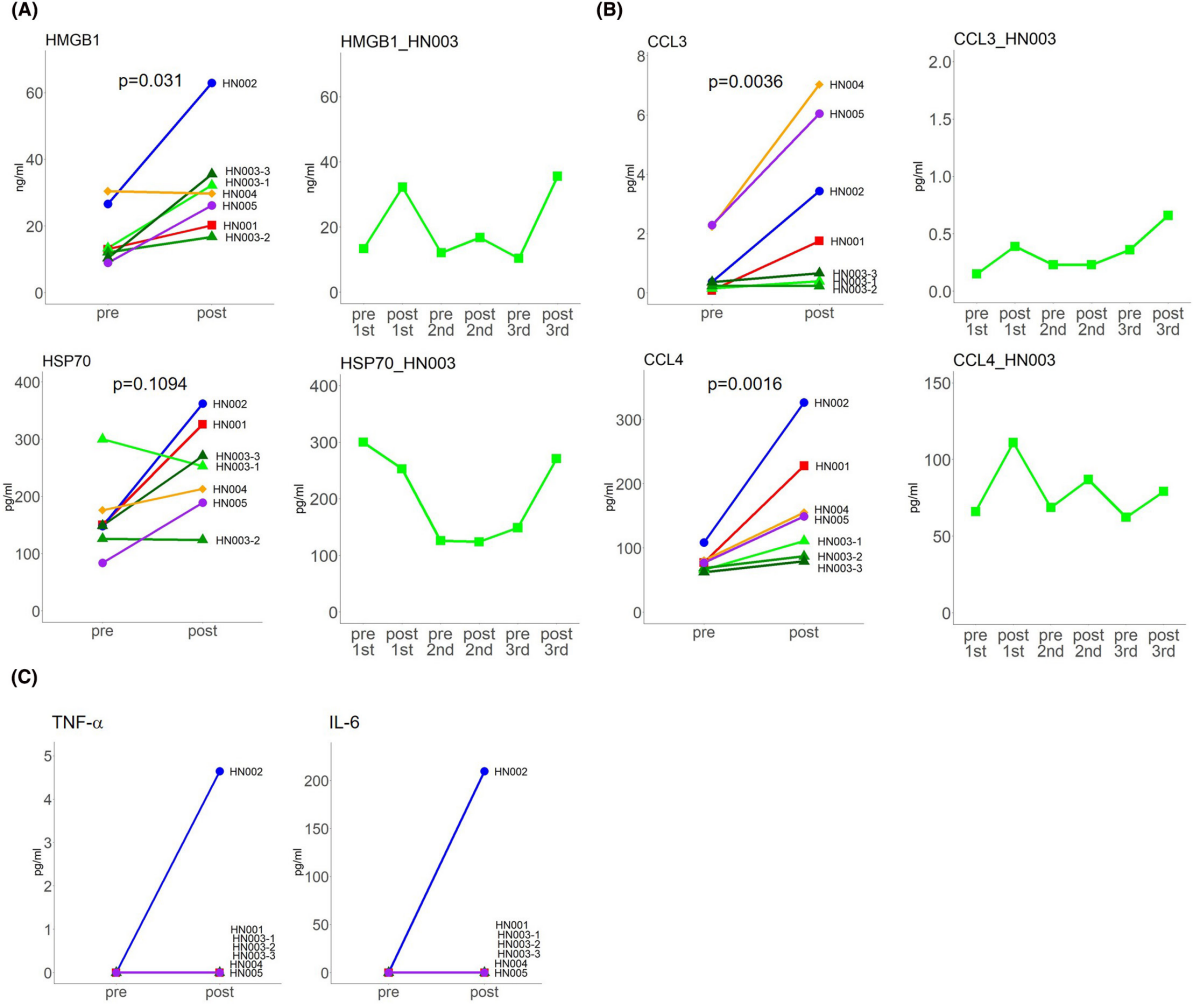
Changes in serum HMGB1 concentration and cytokine/chemokine concentrations before and after NIR‐PIT. (A) Change in serum HMGB1 and Hsp70 concentrations before and after treatment (pre‐, pre‐treatment; post‐, post‐treatment) in a total of seven NIR‐PIT cycles (left). Changes in HMGB1 and Hsp70 concentrations over the course of treatment in patient HN003 (right). (B) Change in the serum concentration of MIP‐1 alpha (CCL3) and MIP‐1 beta (CCL4) before and after treatment in a total of seven NIR‐PIT cycles (left). MIP‐1 alpha (CCL3) and MIP‐1 beta (CCL4) concentration changes over the course of treatment in patient HN003 (right). (C) Changes in the concentration of TNF alpha and IL‐6 in the serum before and after treatment in a total of seven NIR‐PIT cycles.

Five patients with HNSCC were treated with NIR‐PIT (Table 1). Patient HN003 received the treatment three times. To investigate whether DAMPs were induced by NIR‐PIT, the serum concentrations of HMGB1 and Hsp70 were measured before and after therapy (Figure 1A). Significant differences in HMGB1 levels were observed before and after seven cycles of NIR‐PIT treatment (*p* = 0.031, Wilcoxon test) (Figure 1A upper left). The concentration of HMGB1 increased after treatment in all patients except HN004. For patient HN003, the HMGB1 concentration increased after therapy but decreased before subsequent treatment (Figure 1A upper right). In addition, the concentrations of Hsp70 also increased in most patients after treatment (Figure 1A lower left). For patient HN003, the changes in Hsp70 concentration over the course of treatment were similar to those of HMGB1, except before and after the first treatment (Figure 1A lower right).

**TABLE 1:** Clinicopathological features, adverse events and clinical outcomes of five patients treated with NIR‐PIT.

Case	Gender	Age	ECOG PS	TNM	Histology	Primary site	Previous treatment	Adverse events	BOR (best overall response)^a^	OS (months)	PFS (months)	Tumor recurrence after'NIR‐PIT treatment	Outcome
HN001	Male	42	0	rT2N1M0	SCC	Buccal mucosa	Surgical operation	Pain G2, edema G2	CR	16	4	Cervical lymph node	Alive
HN002	Male	87	1	rT4aN0M0	SCC	Lower gingiva	Radiation	Pain G1, edema G4	CR	8	7	Primary lesion	Dead
HN003	Female	74	0	rT1N0M0	SCC	Nasal cavity	Surgical operation	Pain G1	CR	12	12	No recurrence	Alive
HN004	Male	79	1	rT4aN0M0	SCC	Upper gingiva	Surgical operation, post‐operative chemoradiation	Fistula G2	PR	7	0	Tumor remnant	Dead
HN005	Male	75	0	rT2N0M0	SCC	Hypopharynx, oropharynx	Surgical operation, radiation	Edema G2, laryngeal necrosis G3	CR	6	6	No recurrence	Alive

^a^ BOR (best overall response) is based on mRECIST 1.1.

### 3.2 Changes in serum cytokine and chemokine production after NIR‐PIT

Next, we investigated the production of cytokines and chemokines in the sera before and after NIR‐PIT. GM‐CSF, IL‐2, IFN gamma, IL‐4, IL‐12 p70, IL‐1 beta, IL‐5, IL‐13, CXCL1, and CXCL8 were below the detection sensitivity both before and after treatment. In contrast, CCL2, CCL3, CCL4, CCL5, CCL11, CXCL10, CXCL12, IL‐18, and were detected both before and after treatment. Among them, CCL3 and CCL4 levels significantly increased after treatment (CCL3, *p* = 0.0036; CCL4, *p* = 0.0016; Wilcoxon test) (Figure 1B left). In patient HN003, CCL4 increased after treatment in all three cycles and decreased before the subsequent treatment, in parallel with the changes in HMGB1 concentration (Figure 1B lower right). There were no differences in the concentrations of CCL2, CCL5, CXCL10, CCL11, CXCL12, or IL‐18 before and after treatment (Figure S1). Two inflammatory cytokines, TNF‐alpha and IL‐6, were not detected before treatment in all seven NIR‐PIT cycles. However, in patient HN002, we detected elevated levels of these cytokines after therapy (Figure 1C).

### 3.3 Association between changes in HMGB1 and cytokine/chemokine production and clinical response or adverse events

The treatment efficacy, adverse events, and prognosis for all patients are summarized in Table 1. The effectiveness of NIR‐PIT was evaluated using the modified RECIST (mRECIST 1.1^8^) assessment, which is based on a reduction in the size of the target site. However, rapid tumor necrosis observed after treatment makes it difficult to precisely determine the extent of the tumor. In such cases, a negative biopsy results or the absence of tumor growth during follow‐up may warrant a classification as a complete response (CR). Four patients achieved a CR, and the tumor disappeared in the target area. Patient HN004 had a partial response (PR), in which the tumor in the target area shrank. Notably, the patient's HMGB1 levels did not increase after treatment (Figure 1A upper left), suggesting a relationship with treatment resistance. In patient HN002, a prominent grade 4 laryngeal edema appeared after treatment. The detection of TNF alfa and IL‐6 in the peripheral blood serum (Figure 1C) may reflect the result of greater induced inflammation than expected (Table 1).

### 3.4 Pre‐treatment peripheral blood neutrophil‐to‐lymphocyte ratios and their relationship with therapeutic efficacy and prognosis

Finally, we assessed whether the baseline immunological status affected treatment efficacy and patient outcome. White blood cell, lymphocyte, and neutrophil counts, and neutrophil‐to‐lymphocyte ratios (NLR) were measured in the peripheral blood before treatment (Figure S2). The NLR was higher in patients HN002 and HN004. Patients HN002 and HN004 survived 8 months or less after treatment (Table 1).

## 4 DISCUSSION

In the present study, seven NIR‐PIT sessions were administered to five patients with HNSCC. We assessed cancer cell death and immune responses following NIR‐PIT, as well as baseline immunological status, and their relationships with treatment efficacy and adverse events.

NIR‐PIT induces several markers of ICD, potentially activating a sustained immune response.^12^, ^13^ DAMPs include calreticulin (CRT), Hsp70, ATP, HMGB1, etc., the appearance of which on the surface of dying cells or in the tumor microenvironment help determine whether cell death is immunogenic.^14^, ^15^, ^16^ HMGB1 release from tumor cells after chemoradiotherapy was related to better clinical outcomes in patients with esophageal squamous cell carcinoma (ESCC).^17^ In our study, seven NIR‐PIT cycles were administered to five patients with HNSCC, and the release of HMGB1 was detected. No increase in HMGB1 was observed in patient HN004, who showed a poorer treatment response than the others, suggesting an association with a poor treatment response.

It has been known that DAMPs induce dendritic cell (DC) maturation and provoke cytokine release. The addition of HMGB1 to human peripheral blood mononuclear cell cultures in vitro markedly stimulated the release of cytokines such as TNF, IL‐1α, IL‐1β, IL‐1RA, IL‐6, IL‐8, CCL3, and CCL4.^18^ DCs exposed to photoimmunotherapy‐killed FaDu cells had significantly higher concentrations of inflammatory cytokines, including CCL3 and CCL4.^19^ It has been shown that CCR5, a chemokine receptor on naive CD8^+^ T cells, is upregulated in immunogen‐draining lymph nodes, and these cells are attracted to antigen‐specific DC‐CD4 T cell interaction sites, where cognate chemokines CCL3 and CCL4 are produced.^20^ Our study also showed a significant increase in CCL3 and CCL4 after NIR‐PIT, indicating that DAMPs such as HMGB1 and Hsp70 may also stimulate immune cells, such as intratumoral DCs, in patients who received NIR‐PIT. These data may be evidence of ICD following NIR‐PIT; however, we did not clear whether these chemokines originated from DCs after exposure to DAMPs or from inflammatory cells at the site of treatment. In addition, we did not investigate whether adaptive immune responses were induced in the patients.

The NLR has been acknowledged as a predictor of malignancy prognosis and the efficacy of ICIs therapy. A meta‐analysis of NLR as a prognostic marker in patients with HNSCC treated with ICIs showed that a higher NLR was associated with poor overall survival, progression‐free survival, response, and disease control.^21^ In our study, patients HN002 and HN004, who had high NLRs, died within 8 months; patient HN004 had a poorer response to treatment than the others. Therefore, similar to ICIs therapy, the NLR may predict treatment response and survival in patients with NIR‐PIT.

This study has several limitations. First, peripheral blood samples, but not local tumor tissues, were used to evaluate the production of DAMPs and cytokines/chemokines. Peripheral blood samples may not accurately reflect the immune response at the tumor site. Second, we have not investigated an adaptive immune response which is a criterion for ICD. Third, this is a small case series with preliminary observation. Therefore, the results need to be verified in future phase III trials and a significant number of subsequent studies.

## AUTHOR CONTRIBUTIONS

**Hiromasa Ishihara:** Conceptualization (equal); data curation (equal); formal analysis (equal); writing – original draft (equal). **Daisuke Nishikawa:** Conceptualization (equal); funding acquisition (equal); project administration (equal); writing – original draft (equal). **Daisuke Muraoka:** Data curation (equal); formal analysis (equal). **Katsuhiro Masago:** Data curation (equal); formal analysis (equal). **Shintaro Beppu:** Resources (equal). **Hoshino Terada:** Resources (equal). **Hirokazu Matsushita:** Conceptualization (equal); funding acquisition (equal); writing – original draft (equal). **Nobuhiro Hanai:** Conceptualization (equal); project administration (equal); supervision (equal); writing – original draft (equal).

## FUNDING INFORMATION

This work was supported in part by the Japan Society for the Promotion of Science KAKENHI grant numbers 21K09666 (D.N.) and was also supported in part by the Aichi Cancer Center Joint Research Project on Priority Areas (H.M.).

## CONFLICT OF INTEREST STATEMENT

**N.H.** received honoraria (lecture fee) from Rakuten Medical.

## ETHICAL APPROVAL STATEMENT

This study was approved by the Ethics Committees of Aichi Cancer Center (2021–0‐066).

## INFORMED CONSENT STATEMENT

Informed consent was obtained from all subjects involved in the study.

## Supporting information


Figures S1‐S2.

## Data Availability

Data are available upon reasonable request.
