# Supplementary material for: Changes in serum DAMPs and cytokines/chemokines during near‐infrared photoimmunotherapy for patients with head and neck cancer
Source: Cancer Med. 2023 Dec 22;13(1):e6863. doi: 10.1002/cam4.6863 (PMC10807567; doi:10.1002/cam4.6863)
Supplement: Supplementary file 1 — Figures S1‐S2. [file CAM4-13-e6863-s001.pdf]

Figure S1

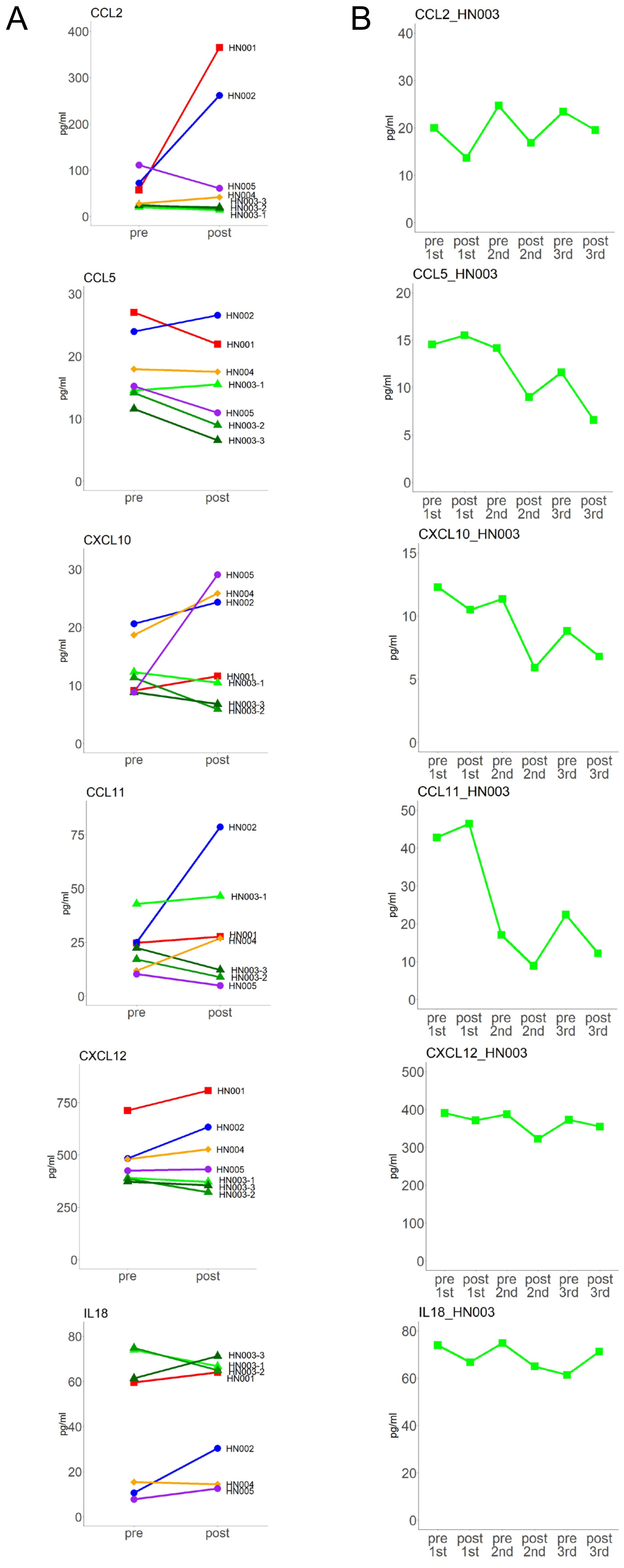

Figure S1. Changes in serum cytokine/chemokine concentration before and after NIR-PIT.
(A) Changes in the serum concentration of MCP-1 (CCL 2), RANTES (CCL5), IP-10 (CXCL10), Eotaxin (CCL11), SDF-1 alpha (CXCL12), and IL-18 before and after treatment (pre, pre-treatment; post, post-treatment) in a total of seven NIR-PIT cycles. (B) Changes in the concentration of the six cytokines/chemokines over the course of treatment in patient HN003.

Figure S2

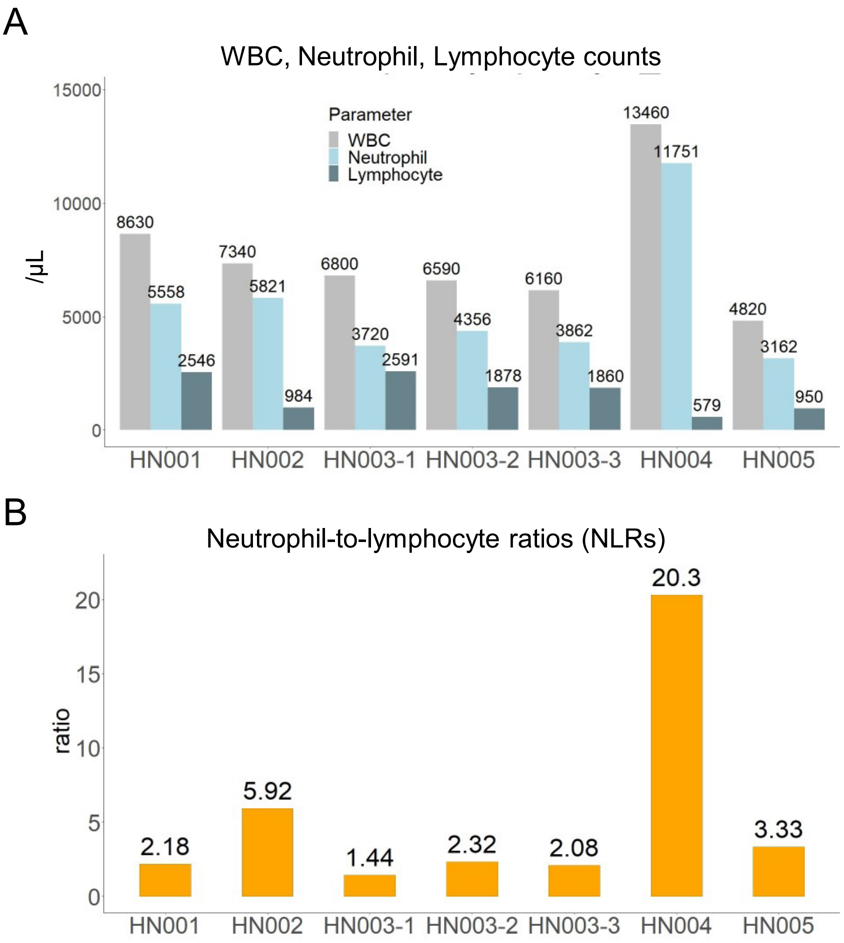

Figure S2. Pre-treatment blood laboratory findings and neutrophil-to-lymphocyte ratios (NLRs). (A) White blood cell, neutrophil, and lymphocyte counts before treatment. (B) NLR of peripheral blood before treatment, with higher values in patients HN002 and HN004.
